# Epigenetic Changes in Neoplastic Mast Cells and Potential Impact in Mastocytosis

**DOI:** 10.3390/ijms22062964

**Published:** 2021-03-15

**Authors:** Edyta Reszka, Ewa Jabłońska, Edyta Wieczorek, Peter Valent, Michel Arock, Gunnar Nilsson, Bogusław Nedoszytko, Marek Niedoszytko

**Affiliations:** 1 Department of Molecular Genetics and Epigenetics, Nofer Institute of Occupational Medicine, 91-348 Lodz, Poland; Ewa.Jablonska@imp.lodz.pl (E.J.); Edyta.Wieczorek@imp.lodz.pl (E.W.); 2 Department of Internal Medicine I, Division of Hematology and Hemostaseology, Medical University of Vienna, 1090 Vienna, Austria; peter.valent@meduniwien.ac.at; 3 Ludwig Boltzmann Institute for Hematology and Oncology, Medical University of Vienna, 1090 Vienna, Austria; 4 Department of Hematological Biology, Pitié-Sapêtrière Hospital, Sorbonne University, 75013 Paris, France; michel.arock@aphp.fr; 5 Division of Immunology and Allergy, Department of Medicine, Karolinska Institutet and Karolinska University Hospital, SE-171 76 Stockholm, Sweden; gunnar.p.nilsson@ki.se; 6 Department of Dermatology, Venereology and Allergology, Medical University of Gdansk, 80-211 Gdansk, Poland; bned@gumed.edu.pl; 7 Invicta Fertility and Reproductive Center, Molecular Laboratory, Polna 64, 81-740 Sopot, Poland; 8 Department of Allergology, Medical University of Gdansk, 80-211 Gdansk, Poland

**Keywords:** mastocytosis, epigenetics, DNA methylation, demethylating agents, *DNMT3A*, *TET2*, microRNA

## Abstract

Systemic mastocytosis (SM) is a hematologic neoplasm with abnormal accumulation of mast cells in various organ systems such as the bone marrow, other visceral organs and skin. So far, only little is known about epigenetic changes contributing to the pathogenesis of SM. In the current article, we provide an overview of epigenetic changes that may occur and be relevant to mastocytosis, including mutations in genes involved in epigenetic processes, such as *TET2*, *DNMT3A* and *ASXL1*, and global and gene-specific methylation patterns in neoplastic cells. Moreover, we discuss methylation-specific pathways and other epigenetic events that may trigger disease progression in mast cell neoplasms. Finally, we discuss epigenetic targets and the effects of epigenetic drugs, such as demethylating agents and BET-targeting drugs, on growth and viability of neoplastic mast cells. The definitive impact of these targets and the efficacy of epigenetic therapies in advanced SM need to be explored in future preclinical studies and clinical trials.

## 1. Clinical Features and Classification of Mastocytosis

Systemic mastocytosis (SM) is a neoplastic disease of the bone marrow (BM) characterized by infiltration and abnormal accumulation of mast cells (MC) in the BM, liver, spleen, lymph nodes, skin and other organs. In a subset of patients, release of mediators from MC results in systemic symptoms, and sometimes even in anaphylaxis [1,2,3,4,5,6]. According to the World Health Organization (WHO) classification, mastocytosis can be divided into the following variants: cutaneous mastocytosis (CM), SM, and local MC tumors. SM is split into five categories with varying prognosis: the most prevalent form is indolent SM (ISM); less frequently detected variants of SM are smoldering SM (SSM), and advanced forms of SM; i.e., SM with an associated hematologic neoplasm (SM-AHN), aggressive SM (ASM) and MC leukemia (MCL) [4,7,8].

Clinical manifestations of mastocytosis are manifold. Several of the symptoms recorded are induced by MC-derived mediators. Other symptoms are caused by MC infiltration in various organs. Main symptoms affecting the patients’ quality of life are symptoms related to skin lesions, anaphylaxis, osteoporosis, gastrointestinal problems, fatigue, anxiety, and depression [1,2,3].

In children and adults, mastocytosis is usually an acquired disease, although rare familial cases have been observed [1,8]. In a majority of younger patients, MC accumulation and infiltration is located primarily or even exclusively in the skin, leading to the diagnosis of CM. In many of these children, CM regresses at puberty. Indolent and advanced forms of SM are very rare in children, but are the dominant form of mastocytosis in adults. In SM variants, the detection or exclusion of B- and/or C-Findings is crucial for the proper classification and treatment. B-Findings indicate a huge MC burden: these B-Findings include (1) BM biopsy showing >30% infiltration by MC (focal dense aggregates) and tryptase level above 200 ng/mL (2) signs of dysplasia or myeloproliferation in non-MC lineages but insufficient to diagnose SM-AHN, (3) hepatomegaly and/or splenomegaly without hypersplenism and/or lymphadenopathy on palpation or imaging [9]. C-Findings describe a clinically significant impairment of organ function (organ damage) caused by the MC infiltrates: (1) BM dysfunction with one or more cytopenia(s) (absolute neutrophil count = ANC < 1.0 × 10^9^/L, Hgb < 10 g/dL, or platelets < 100 × 10^9^/L, (2) hepatomegaly with impairment of liver function, ascites and/or portal hypertension, (3) skeletal involvement with large osteolytic lesions and pathological fractures, (4) palpable splenomegaly with hypersplenism, (5) malabsorption with weight loss [4,5,8]. The presence of one or more C-Finding(s) leads to the diagnosis of advanced SM: ASM, SM-AHN, or acute MCL [5,8,10]. While patients with CM or ISM have an excellent prognosis, patients with ASM, SM-AHN or MCL have a poor or very poor prognosis as their disease is often resistant to various treatments [11,12]. 

### KIT Mutations

Stem cell factor (SCF)-induced KIT activation results in MC differentiation from their progenitor cells as well as MC survival and MC maturation [13,14,15]. At higher concentrations, SCF can also augment IgE-dependent mediator release from MC [13,14,15]. KIT (CD117) belongs to the type III receptor tyrosine kinase family and it is encoded by the *KIT* proto-oncogene. An aspartic acid to valine substitution (D816V) in exon 17 of 21 exons, which encodes a second catalytic domain, leads to autonomous activation of KIT and thus to enhanced MC differentiation and survival in the absence of SCF [16,17,18]. This mutation is found in >80% of all adult patients with SM, and thus, this gain-of-function mutation in *KIT* is considered to contribute to the development of SM [12,19]. However, other mutations in *KIT*, both at codon 816 or in other codons of *KIT* (for example 560) have also been reported. Familial mastocytosis has also been described to be associated with *KIT* variants: in these cases, other mutations in *KIT*, such as K509I, may be detected [20].

More than 80% of the observed *KIT* D816V point mutations have been associated with neoplastic phenotype of MC [20]. Interestingly, other *KIT* mutations, e.g., V560G (exon 11), K642E (exon 13) and Y269C (exon 5) have been found in patients with mastocytosis. The *KIT* mutation V560G is also expressed in the human HMC-1 cell line which has been established from the peripheral blood of a patient with MCL [21].

## 2. Epigenetic Changes in Mastocytosis

Epigenetics refers to mechanisms connecting the genetic background with environmental signals in order to provide adaptations to various conditions and threats, like aging, lifestyle factors and also intrinsic pathologic processes [22]. For example, physiologic cell functions and genomic stability are regulated by maintenance of biochemical processes regulated by the epigenome [23]. Epigenetic alterations contribute to the regulation of gene product expression independent of the nucleotide sequence in DNA [24]. Both heritable as well as non-heritable changes can lead to epigenetic modifications. There are different levels of epigenetic regulation that include DNA methylation, posttranscriptional modifications by non-coding RNAs (ncRNAs), histone modifications and nucleosomes positioning, consequently affecting DNA and chromatin structure, respectively [25]. Somatic mutations in critical target genes regulating DNA methylation, chromatin shape and other nuclear processes may also act as epigenetic modifiers.

Unfortunately, very few studies have investigated the role of epigenetic modifications in neoplastic cells in SM. In the current study, we present an overview and update of epigenetic changes described in the context of mastocytosis so far: (1) Histone modifications in neoplastic MC, (2) Epigenetic modifications of *KIT*, (3) Methylation status of apoptosis-associated and tumor suppressor genes in HMC-1 cells, (4) Somatic mutations in genes regulating epigenetic mechanisms, (5) *TET2*, (6) *DNMT3A*, (7) 5-methylcytosine (5-mC), 5-hydroxymethylcytosine (5-hmC) level, (8) microRNA, (9) *MITF* and microRNA.

### 2.1. Histone Modifications in Neoplastic MC

A study by Martinelli et al. revealed that in advanced SM, specific histone modifications can be detected in neoplastic MC in SM, and the same was found in HMC-1 cells [17]. Several histone-modifying proteins were found to be expressed at lower levels in neoplastic MC compared to control cells. One of these proteins is the SET domain containing 2 histone lysine methyltransferase (SETD2) protein [17]. In fact, SETD2 showed reduced expression in patients with MCL. SETD2 is specific for lysine (K) -36 of histone H3, and methylation of this residue is associated with active chromatin. In the study by Martinelli et al., a decrease in H3K36Me3 methylation was correlated with SETD2 protein down-regulation in neoplastic MC in patients with MCL. Particularly, SETD2 loss of function may occur post-translationally, in the absence of mutations and other structural aberrations [26]. Loss of SETD2 function resulted in H3K36 trimethylation deficiency observed in human acute leukemia [27]. The interesting mechanism of epigenetic regulation is related with influence of tryptase on nuclei of MC. Tryptase possesses ability to cleave core histones at their N-terminal ends, leading to regulation of chromatin structure [28]. This part of histone is crucial for posttranslational modifications that have a wide epigenetic impact [29]. Tryptase clips nucleosomal histone 3 and histone 2B. Absence of tryptase leads to age-dependent accumulation of lysine 5-acetylated H2B which affects cell phenotype, up-regulation of markers of non-MC lineages and morphological alterations. The results prove that clipping of H2B by tryptase is a novel epigenetic mechanism and an example of intracellular action of tryptase [29]. Studies by Krajewski et al. showed that epigenetic regulation of MC activation during immune response may be caused by altered histone acetylation [30]. They showed that epigenetic properties of curcumin may suppress MC activation. Furthermore, trichostatin, a histone deacetylase inhibitor, may alter cytokine secretion (IL-4, IL-6, TNF-α, IL-13), and decrease expression of IL-33 by IgE-activated BM MC. In addition, continuous exposure with trichostatin led to MC apoptosis [28].

### 2.2. Epigenetic Modifications of KIT

It has turned out recently that chromatin epigenetic silencing might be one of the key regulators of mutated *KIT* inactivation [31]. However, the specific mechanism of action requires further investigation. Lyberg at al. in 2017 showed that epigenetic treatment with the histone deacetylase inhibitor (HDACi) suberoyl anilide hydroxamid acid (SAHA), is especially effective in *KIT* D816V-mutated BM MC and HMC-1 cells [32]. Among the applied HDACi, panobinostat, romidepsin and valproic acid, SAHA treatment rapidly increased total acetylated histones, followed by a decrease in phosphorylated KIT in the *KIT*-mutated human MC lines HMC-1 and ROSA (*KIT* D816V). Using ChIP qPCR resulted in a decreased active chromatin mark H3K18ac/H3 only in the *KIT* region but not in control genes upstream and downstream of *KIT* (PDGFRα and KDR). MC exposed to SAHA showed downregulation of *KIT* mRNA expression followed by decreased KIT protein expression, and apoptosis. Interestingly, primary MC isolated from SM patients, including one with MCL, were highly sensitive to SAHA-mediated cell death, while normal BM cells were resistant [32].

### 2.3. Methylation Status of Apoptosis-Associated and Tumor Suppressor Genes in HMC-1 Cells

Ghanim et al. explored the methylation status of 24 classical apoptosis-associated genes and 24 classical tumor suppressor genes (TSG) in HMC-1 cells and in control (normal BM) cells [31]. Their data showed that several of them were hypermethylated in the neoplastic MC line. Seven apoptosis-associated genes (*CIDEB*, *GADD45A*, *HRK*, *TNFRSF25*, *BIK*, *BID* and *TNFRSF21*) and six TSG (*NEUROG1*, *CDH1*, *GSTP1*, *CDH13*, *TP73* and *WIF1*) were found to be methylated in HMC-1 cells but not in normal BM cells, suggesting that these genes are aberrantly hypermethylated in these neoplastic MC.

### 2.4. Somatic Mutations in Genes Regulating Epigenetic Mechanisms

Clinical presentations of the disease vary widely from indolent to advanced forms, and to the exceedingly rare MCL. *KIT* D816V mutation solely does not explain heterogeneity of SM with different disease phenotypes. Thus, extensive mutational analyses of patients may provide a better explanation of mastocytosis pathogenesis. Interestingly, several somatic mutations have been observed in epigenetically relevant genes.

There is still scarce research on non-*KIT* epigenetic-associated mutations in various types of mastocytosis. Genes related to epigenetic modification that have been found to be mutated in specific SM types are *TET2* (TET methylcytosine dioxygenase 2), *DNMT3A* (DNA methyltransferase 3 alpha), *ASXL1* (ASXL transcriptional regulator 1), *EZH2* (enhancer of zeste homolog 2) and *IDH1/2* (isocitrate dehydrogenase 1/2) (Table 1).

In five studies on patients with different types of SM (Table 2), a panel of mutated non-*KIT* genes has been investigated. Bone marrow or peripheral blood were used as a source of target DNA with various sequencing applications [18,33,34,35].

Non-*KIT* mutations were less frequent but still comprised a substantial proportion of the mastocytosis patients. In the pilot study, the coding regions of 22 genes were investigated in 19 advanced SM patients. Overall, *KIT* D816V frequency was 89%, while non-*KIT* mutation was observed in fourteen patients (74%) who carried at least one additional mutation. The highest prevalence occurred for *ASXL1/TET2* (*n* = 5, 26%), *CBL* (4 patients, 21%), *SETBP1/SF3B1/DNMT3A* (*n* = 2, 11%) and *JAK2/CALR/FLT3/IDH-1/RUNX1/TP53* (*n* = 1, 5%) [17]. In a study of 150 ASM cases, Pardanani et al. observed a significantly different distribution of non-*KIT* mutations within 27 investigated gene targets in four SM-AHN groups, MCL and ISM. In the whole group of SM patients, the most frequent mutations have been observed for *TET2* (29%), *ASXL1* (17%) and *CBL* (11%) [33].

Additional molecular aberrations have been analyzed in a study of 39 patients with *KIT* D816V and various SM variants: ISM (*n* = 10), SSM (*n* = 2), SM-AHN (*n* = 5) and ASM (*n* = 15) or MCL (*n* = 7) with (*n* = 18) or without (*n* = 4) SM. Next-generation sequencing was applied to investigate mutational hotspot regions, and complete exon regions of *EZH2*, *ETV6*, *RUNX1*, and *TET2* were analyzed [34]. A total of 17 mutations (13 frameshift, 2 nonsense and 2 missense) were documented in 2 (15%) of the 13 ISM patients examined, including 2/5 ASM patients (40%), and 8/23 patients with SM-AHN (35%) [35]. In addition, 9 missense, 4 nonsense, 13 frameshift mutations and one in-frame deletion, as well as one splice site mutation have been identified in *TET2* in 15/39 (39%) SM patients. Ten of 15 (67%) patients carried more than one *TET2* mutation. Eight *ASXL1* mutations have been identified in 8 patients with 7 frameshift and 1 nonsense mutation. A missense mutation and 2 splice site mutations have been detected in *EZH2* [34].

The overall survival (OS) was significantly shorter in patients with additional aberrations as compared to those with *KIT* D816V alone [34]. Occurrence of 3 or more non-*KIT* mutations led to reduced OS (*n* = 4, 7 months) compared with fewer than 3 mutations (48 months) [17]. Interestingly, *TET2*, *DNMT3A* and *ASXL1* mutations may affect prognosis, as demonstrated by worse OS in mutated patients. For example, mutation in epigenetic regulators, namely *TET2* and/or *ASXL1*, was associated with reduced OS (11 months) compared with the absence of these mutations (84 months) [17]. In addition, significant differences in OS between patients with *KIT*-mutated SM and patients with combined *TET2/DNMT3A/ASXL1* mutations independent of *KIT* and sole *TET2* mutations (*p* < 0.001) have been observed [36]. To add, in SM-AHN patients *ASXL1*, but not *TET2*, mutation was associated with OS, suggesting different epigenetic changes in heterogeneous SM prognosis [37].

Importantly, mutations in *TET2*, *DNMT3A*, and *ASXL1*, *EZH2* and *IDH1/IDH2* have been observed more frequently in advanced SM, e.g., ASM, SM-AHN and MCL than in ISM patients (Table 2). Mutations in epigenetic driver targets in four groups of SM patients have been observed with the following relative frequencies: SM-AHN > ASM > ISM. In the whole group of SM patients, the most frequent mutations have been observed in *TET2* (29%), *ASXL1* (17%), and *DNMT3A* (6%). The only additional mutations observed in ISM patients are age-related somatic mutations that have also been associated with epigenetic changes, e.g., mutations in *TET2* (7%) and *DNMT3A* (5%) [33]. Thus, mutations in three genes: *TET2*, *DNMT3A*, and *ASXL1*, may be associated with epigenetic features in SM. [36]. However, it remains unknown whether these mutations indeed lead to abnormal methylation patterns or other critical epigenetic changes in clonal cells and, if so, whether these changes indeed contribute to disease evolution and/or the aggressiveness of SM.

Patients with SM-AHN presented mutations in *KIT*, *TET2*, *ASXL1* and *CBL* in 87%, 27%, 14%, and 11%, respectively. In these patients, mutated *ASXL1* had a particularly adverse impact on OS [37].

Mutations in the discussed epigenetic modulators frequently occur in other hematological diseases. Recurrent *TET2*, *DNMT3A*, *IDH1/2* mutations have been observed in patients with myeloproliferative neoplasms and other hematological malignancies, such as acute myeloid leukemia (AML), chronic myelomonocytic leukemia (CMML), or myelodysplastic syndromes (MDS) [38,39]. Thus, two questions can be raised: are these point mutations prognostic in SM and do these mutations have an impact on the responsiveness of neoplastic MC to various targeted drugs.

### 2.5. TET2

Ten-Eleven-Translocation proteins (with 3 isoforms) are enzymes dependent on a-ketoglutarate and Fe^2+^ TET2 is important in demethylation of 5-mC forming 5-hmC by oxidation that enables dynamic turnover of 5mc and 5hmC. In addition, mice with *Tet2* knockdown presented late-onset hematological abnormalities. TET proteins and oxidized methylcytosines are implicated in several pathways of DNA demethylation. They are involved in (1) removal of 5-formylcytosine (5-fC) and 5-carboxylcytosine (5-caC) by thymine DNA glycosylase (TDG), and (2) inhibition of DNA methyltransferase (DNMT)1-mediated maintenance of DNA methylation [40].

Alterations of the numbers, location and function of MC are frequently observed in patients with SM [41]. Mutations in *TET2* and other critical target genes are often detected in primary neoplastic cells in patients with advanced SM, suggesting a possible role of the gene products in abnormal MC accumulation and function in these neoplasms (see Table 2). For example, loss of *Tet2* associated with *Kit* D816V mutation has been related to a transformation of the MC disease into a more aggressive phenotype in mice [42].

Recently, Montagner et al. found that *TET2* might be a crucial regulator of MC proliferation and differentiation, and also of cytokine production [42]. MC lacking *TET2* revealed disrupted gene expression, profound down-regulation of many genomic regions—enhancers, and reduced global 5-hmC deposition, delay in differentiation, defects in cytokine production and a marked increase in proliferation. Interestingly, while cell differentiation under *TET2* absence has been found to be regulated with compensatory mechanisms mediated by *TET1* and *TET3* family members, MC proliferation was only dependent on *TET2* without a compensatory effect by other *TET* family members [43].

### 2.6. DNMT3

Three DNMT enzymes have been described. DNMT1 is responsible for maintenance of DNA methylation, which recognizes hemimethylated CpG sites and replicates DNA methylation pattern in newly synthetized nucleotides in the cell. DNMT3A and DNMT3B are responsible for de novo DNA methylation [25].

DNA methylation and specifically DNA methyltransferase enzyme DNMT3A, involved in de novo methylation, modulates pathogenesis of various hematological diseases and is involved in regulating the function of immune cells. The role of DNA methylation and DNMT3A in MC regulation requires further investigations.

Moreover, it has been observed that DNMT3A is essential for hematopoietic stem cells (HSC) differentiation. HSC lacking *Dnmt3a* presented a level of global 5-mC levels comparable with control cells [44] and loss of *Dnmt3a* immortalized these cells in vivo [45]. Therefore, *Dnmt3a* seems to be critical in the epigenetic silencing of regulatory genes, thereby enabling efficient differentiation of HSC. Interestingly, in murine MC, overall levels of genomic 5-mC were not significantly different in both *Dnmt3a*- knockout and wildtype cells [46].

### 2.7. 5-mC, 5-hmC Level

The only study on global DNA methylation indices, like 5-hmC and 5-mC levels in patients with SM, has revealed that the correlation between SM diagnosis and significantly low 5-hmC levels may represent a systemic effect of SM. Overall reduced 5-hmC levels in all patients with SM have been reported. In SM, reduced level of 5-hmC was associated with the effect of *KIT* D816V mutated oncogene. Neither the loss of 5-hmC correlated with overall MC burden in those patients, nor was it due to inactivating mutations of *TET2* or reduced *TET2* expression [47]

### 2.8. MicroRNA

Non-coding RNA transcripts (ncRNAs), constituting almost 98% of human genome, have been demonstrated to play a significant role in various pathological processes. It is regarded that microRNAs (miRNAs) possess capacity to act as putative negative regulators of gene expression at transcriptional, posttranscriptional and epigenetic levels of the target genes [48].

miRNAs are recognized to be critical regulators of immune responses in various allergic diseases and are involved in cell differentiation, proliferation, survival and IgE-mediated allergic response [49]. In addition, specific miRNAs might be important for mouse MC differentiation. The analysis of expression of 181 mature miRNAs in selected highly purified hematopoietic murine cell types at immature, mature, and effector stages has revealed specific differences between the related cell types [50].

Additionally, in murine MC, miR-221-222 (miR-221 and its paralogue miR-222) has occurred to be significantly up-regulated upon cell activation. Overexpression of miR-221-222 resulted in altered cell morphology and cell cycle regulation without altering viability. Overall, this miRNA was involved in cell cycle regulation in MC in response to acute activation stimuli [51].

Currently, there is excessive evidence that exosome might provide cell-to-cell communication. In 2007, Valadi et al. showed that exosomes derived from a mouse and a human MC line (MC/9 and HMC-1, respectively), and primary bone marrow-derived mouse MC, contained RNA, both microRNA and mRNA. MC-derived RNAs can be further delivered to another cell, and can be functional in this new location. The authors have thus proposed that this RNA could be called “exosomal shuttle RNA” [52].

Characteristics of detailed RNA content have been determined in human MC line, HMC-1 using the microarray assay and miRCURY^TM^ RNA array analysis. Note that 116 microRNAs were found in exosomes and 134 were identified in donor MC, the most abundant microRNAs in the exosomes, compared to the MC were: hsa-miR-451, hsa-miR-503, miRPlus_27560, miRPlus_2843, miRPlus_27564, hsa-miR-583, miRPlus_1795, miRPlus_17890, hsa-miR-663 and hsa-miR-30b. Predictions according to canonical pathways and networks showed involvement of MC-derived exosomal microRNA in cellular development, cellular movement, hematological system development and function as well as in cell cycle regulation [53].

However, it remains unknown whether all these miR species play a role in human (neoplastic) MC and it also remains unknown whether such miR molecules contribute to the pathogenesis of SM.

### 2.9. MITF and MicroRNA

A protein encoded by *MITF* is a transcription factor that contains both basic helix-loop-helix and leucine zipper structural features. The encoded protein regulates melanocyte development and is responsible for pigment cell-specific transcription of the melanogenesis enzyme genes [53]. The transcription factor, microphthalmia-associated transcription factor (MITF) is critical for MC function and it is closely related to the regulation of KIT expression and activity. MITF may also be required for the neoplastic phenotype of MC in the SM context. It has been observed that MITF-deficient MC have down-regulated KIT expression, suggesting that KIT may be a target of MITF [54,55]. MITF has also been found to be highly expressed in neoplastic cells in BM biopsies in 9 of 10 patients with SM carrying *KIT* D816V. A study of Lee et al. indicated that MC with activating *KIT* mutation require MITF expression for MC proliferation [56].

Interestingly, it has been observed that miRNA regulation can link these two factors essential for MC function. Lee at al. have revealed that miR-539 and miR-381 are repressed by KIT D816V signaling through conserved miRNA binding sites in the MITF 3’-untranslated region. Normally, expression of these miRNAs is involved in MITF suppression and inhibition of colony-forming capacity of mastocytosis cell lines. Importantly, dysregulation of this pathway may contribute to abnormal MC proliferation and thus to the pathogenesis of aggressive MC diseases, both in humans and in the murine system [56].

Studies on the regulation of miRNA with other epigenetic key players are still at an initial stage. For example, findings from acute myeloid leukemia cells have indicated strong impact of miR-29 family members on three target DNMT izoenzymes: DNMT1, DNMT3A and DNMT3B resulting in global DNA hypomethylation as well as reexpression of tumor suppressor genes [57].

In the study on murine MC, although primary screening has not revealed modification of knockout *Dnmt3a* MC by any microRNA, it has been observed that miR-223 was dysregulated in cells lacking *Dnmt3a* [46]. Additionally, degranulation of MC followed by down-regulation of miR-223 has been observed in IgE-activated cells [58], suggesting the importance of miR-223 in epigenetic regulation of MC regardless of a MC immune response or differentiation.

## 3. Effects of Epigenetic Drugs on Growth and Viability of Neoplastic MC

The classical epigenetic drugs are the hypomethylating agents, such as azacytidine and decitabine. Both drugs are highly active in patients with advanced MDS, CMML and AML [59,60]. So far, however, only a few studies have examined the potential effects of these epigenetic drugs in the SM context or in SM-AHN.

A study of Ghanim et al. examined the antineoplastic effects of demethylating agents on human neoplastic MC. They found that the death regulator FAS is hypermethylated in neoplastic MC, but not in normal BM cells, and that azacytidine and decitabine can induce demethylation and thereby can promote expression of *FAS* mRNA, *p21* mRNA, *Noxa* mRNA, and *Bim* mRNA in neoplastic MC [61].

Another potential epigenetic target in neoplastic MC is the epigenetic reader BRD4 which regulates expression of MYC in normal and neoplastic cells, including AML cells [62]. Wedeh et al. have shown that neoplastic MC in SM, including advanced SM, display BRD4 and MYC. The MC lines HMC-1 and ROSA also express BRD4 and MYC at the mRNA and protein level. Moreover, the BRD4-targeting drug JQ1 was found to induce dose-dependent growth inhibition and apoptosis in HMC-1 cells and ROSA cells and to suppress proliferation of primary neoplastic MC obtained from patients with ASM or MCL [63].

More recently, SETD2 histone methyltransferase was identified as a potential target in advanced SM. Indeed, Martinelli et al. have reported that very low or absent SETD2 protein expression was especially found in cases with advanced SM, and proteasomal degradation was found to have a major role in the observed lack this protein. Inhibitors of the proteasome, such as bortezomib, can rescue (the epigenetically decreased) expression of SETD2 and H3K36 trimethylation in neoplastic MC, resulting in a marked accumulation of ubiquitinated SETD2. Bortezomib was also found to counteract viability in neoplastic MC and to cooperate with midostaurin in inducing apoptosis in HMC-1 cells and neoplastic MC from patients with advanced SM [26].

The use of epigenetic drugs may pave the way for the development of improved treatment approaches in advanced SM. However, more preclinical studies and clinical trials need to be performed to confirm the impact of epigenetic targets and the efficacy of epigenetic therapies in patients with MC neoplasms.

## 4. Concluding Remarks

Mastocytosis is a group of heterogeneous diseases with a broad range of molecular patterns and markers and a variable clinical course. Several disease-specific processes may be triggered by the epigenome in these patients. However, only little is known about the role of epigenetic modifications in neoplastic cells in SM.

The current paper provides a summary of recent findings regarding epigenetic changes in neoplastic cells in various types of mastocytosis. In general, data published recently suggest that epigenetic changes may indeed trigger the pathogenesis of SM through several different mechanisms, including aberrant methylation of promoters of genes critically involved in DNA/RNA processing, apoptosis, and activation of MC, and modulation of KIT expression by epigenetic regulators. Moreover, genes whose products influence epigenetic processes including *TET2*, *DNMT3A* or *ASXL1* may be altered (mutated) in mastocytosis. *Such* mutations are frequently observed in advanced SM and these mutations may also affect clinical behavior of SM and thus prognosis. In addition, *TET2* and *DNMT3A may* play a role in MC responsiveness to acute and chronic activation. These observations suggest a potential influence of epigenetic processes on the pathogenesis of MC-related disorders.

## Figures and Tables

**Table 1:** Mutated genes related to epigenetic modification in mastocytosis ^1^.

Gene (*H. sapiens*)	Gene ID	Protein Product	Protein Function and Biological Importance	Expression	Location, Exon Count
*TET2*	54790	TET methylcytosine dioxygenase 2	catalyzes the conversion of methylcytosine to 5-hydroxymethylcytosine. The encoded protein is involved in myelopoiesis, and defects in this gene have been associated with several myeloproliferative disorders	ubiquitous expression in bone marrow, appendix and 25 other tissues	4q24 15
*ASXL1*	171023	ASXL transcriptional regulator 1	ligand-dependent co-activator for retinoic acid receptor in cooperation with nuclear receptor coactivator 1. Mutations in this gene are associated with MDS and CMML	ubiquitous expression in testis, lymph node and 25 other tissues	20q11.21 18
*DNMT3A*	1788	DNA methyltransferase 3 alpha	de novo methylation, localizes to the cytoplasm and nucleus and its expression is developmentally regulated	ubiquitous expression in placenta, ovary and 25 other tissues	2p23.3 34
*IDH2*	3418	isocitrate dehydrogenase (NADP(+)) 2	catalyzes the oxidative decarboxylation of isocitrate to 2-oxoglutarate; localized in mitochondria, plays a role in intermediary metabolism and energy production. This protein may tightly associate or interact with the pyruvate dehydrogenase complex	ubiquitous expression in heart, kidney and 24 other tissues	15q26.1 12
*EZH2*	2146	enhancer of zeste 2 polycomb repressive complex 2 subunit	involved in maintaining the transcriptional repressive state of genes over successive cell generations	broad expression in bone marrow, testis and 14 other tissues	7q36.1 25

^1^ NCBI: https://www.ncbi.nlm.nih.gov/ (accessed on 10 March 2021).

**Table 2:** non-*KIT* and epigenetic mutations in different variants of mastocytosis.

Genes with No. of Mutations/No. of Patients (%)				Samples; Molecular Screening	Ref.
All	ISM	ASM	SM-AHN		
*TET2* 12/42 (29)	*TET2* 2/13 (15)	*TET2* 2/5 (40)		BM; bidirectional sequences ^1^	[35]
*TET2* 44/150 (29)	*TET2* 1/15 (7)			BM and PB; direct genomic sequences ^2^	[36]
*ASXL1* 25/150 (17)	*ASXL1* 1/15 (7)				
*DNMT3A* 9/150 (6)	*DNMT3A* 2/15 (13)				
EZH2 0	*EZH2* 0				
IDH1/2 0	*IDH1/2* 0				
*TET2* 15/39 (38)				BM and PB; NGS, 18 genes ^3^	[34]
*ASXL1* 8/39 (21)					
*EZH2* 2/39 (5)					
*TET2* 5/19 (26)		*TET2* 1/6 (2)	*TET2* 4/13 (31)	BM; NGS, 22 genes	[17]
*ASXL1* 5/19 (26)		*ASXL1* 1/6 (2)	*ASXL1* 4/13 (31)		
*DNMT3A* 2/19 (10)		*DNMT3A* 0	*DNMT3A* 2/13 (15)		
*TET2* 44/150 (29)	*TET2* 3/44 (7)	*TET2* 5/25 (20)	T*ET2* 36/80 (45)	BM; NGS, 27 genes ^4^	[33]
*ASXL1* 25/150 (17)	*ASXL1* 0	*ASXL1* 4/25 (16)	*ASXL1* 21/80 (26)		
*DNMT3A* 9/150 (6)	*DNMT3A* 2/44 (5)	*DNMT3A* 0	*DNMT3A* 7/80 (9)		
*EZH2* 3/150 (2)	*EZH2* 0	*EZH2* 1/25 (4)	*EZH2* 2/80 (3)		
*IDH1/2* 4/150 (3)	*IDH1/2* 0	*IDH1/2* 1/25 (4)	*IDH1/2* 3/80 (4)		

ISM, indolent systemic mastocytosis; ASM, aggressive SM; SM-AHN, SM with associated hematologic neoplasm; PB, peripheral blood; BM, bone marrow; ^1^ *TET2* (all exons); ^2^ *TET2* (all exons), *DNMT3A* (all exons), *ASXL1* (exon 12), *EZH2* (all exons), *IDH1* (exon 4), *IDH2* (exon 4), SM-AHN, aggressive mastocytosis and MC sarcoma included; ^3^ *TET2* (all exons), *EZH2* (all exons), *ASXL1* (exon 12) by Sanger sequencing, SM, SM-AHN included; ^4^ MCL included.

## Data Availability

Not applicable.

## Abbreviations

AML	Acute Myeloid Leukemia
ASM	aggressive systemic mastocytosis
ASXL1	additional sex combs like 1
BET	Bromodomain and Extra-Terminal Motif
CALR	calreticulin
CBL	casitas B-cell lymphoma
CEBPA	CCAAT/enhancer-binding protein-alpha
CM	cutaneous mastocytosis
CMML	chronic myelomonocytic leukemia
DNMT1	DNA methyl transferase 1
DNMT3A	DNA methyltransferase 3 alpha
DNMT3B	DNA methyltransferase -3B
ECM	extracutaneous mastocytoma
ETV6	ets family transcription factor
EZH2	enhancer of zeste homolog 2
FLT3	Fms Related Tyrosine Kinase
HDACi	histone deacetylase inhibitor
HSC	hematopoietic stem cells
IDH1/2	isocitrate dehydrogenase 1/2
IgE	immunoglobulin E
ISM	indolent systemic mastocytosis
JAK2	Janus Kinase-2
MCL	mast cell leukemia
MDS	myelodysplastic syndrome
miRNAs	micro RNAs
MITF	microphthalmia-associated transcription factor
MLL-PTD	partial tandem duplication of MLL
ncRNA	non-coding RNA
NPM1	nucleophosmin 1
NRAS	neuroblastoma rat sarcoma viral oncogene homolog
PTPN11	protein tyrosine phosphatase non-receptor type 11
OS	overall survival
RUNX1	runt-related transcription factor 1
SAHA	suberoyl anilide hydroxamid acid
SETBP1	SET binding protein 1
SM	systemic mastocytosis
SM-AHN	systemic mastocytosis with an associated hematologic neoplasm
SRSF2	serine/arginine-rich splicing factor 2
SSM	smouldering systemic mastocytosis
SUZ12	suppressor of zeste 12 homolog
TDG	thymine DNA glycosylase
TET2	Ten-Eleven-Translocation 2
U2AF1	U2 auxiliary factor 1
5-caC	5-carboxylcytosine
5-fC	5-formylcytosine
5-hmC	5-hydroxymethylcytosine
5-mC	5-methylcytosine
